# Handheld Multifunctional Fluorescence Imager for Non-invasive Plant Phenotyping

**DOI:** 10.3389/fpls.2022.822634

**Published:** 2022-04-08

**Authors:** Ruochong Zhang, Sally Shuxian Koh, Mark Ju Teng Teo, Renzhe Bi, Shuyan Zhang, Kapil Dev, Daisuke Urano, U. S. Dinish, Malini Olivo

**Affiliations:** ^1^Translational Biophotonic Laboratory, Institute of Bioengineering and Bioimaging, A*STAR, Singapore, Singapore; ^2^Temasek Life Sciences Laboratory, Singapore, Singapore; ^3^Department of Biological Sciences, National University of Singapore, Singapore, Singapore

**Keywords:** fluorescence, multifunctional, handheld, non-invasive plant phenotyping, photosynthetic activity, anthocyanin

## Abstract

Fluorescence imaging has shown great potential in non-invasive plant monitoring and analysis. However, current systems have several limitations, such as bulky size, high cost, contact measurement, and lack of multifunctionality, which may hinder its applications in a wide range of settings including indoor vertical farming. Herein, we developed a compact handheld fluorescence imager enabling multipurpose plant phenotyping, such as continuous photosynthetic activity monitoring and non-destructive anthocyanin quantification. The compact imager comprises of pulse-amplitude-modulated multi-color light emitting diodes (LEDs), optimized light illumination and collection, dedicated driver circuit board, miniaturized charge-coupled device camera, and associated image analytics. Experiments conducted in drought stressed lettuce proved that the novel imager could quantitatively evaluate the plant stress by the non-invasive measurement of photosynthetic activity efficiency. Moreover, a non-invasive and fast quantification of anthocyanins in green and red Batavia lettuce leaves had excellent correlation (>84%) with conventional destructive biochemical analysis. Preliminary experimental results emphasize the high throughput monitoring capability and multifunctionality of our novel handheld fluorescence imager, indicating its tremendous potential in modern agriculture.

## Introduction

Effective plant monitoring techniques are critical in gaining insights on plant health, allowing for improved decision-making and a more streamlined workflow in modern agriculture. Most land plants produce their own food by chlorophyll, a group of green pigments. Part of the ambient light absorbed by chlorophyll are used to produce carbohydrates through photochemistry and the unused parts are either dissipated as heat or re-emitted as longer wavelength fluorescence. These three processes are in competition and chlorophyll fluorescence emission was found to be dynamic and complex with a plenty of information (Kautsky, 1960). Hence, chlorophyll fluorescence has been widely used as an indicator of photosynthetic activity (PSA) and plant self-protection mechanism. Modulated chlorophyll fluorescence measurement methods (Quick and Horton, 1984; Schreiber et al., 1986) became more popular than non-modulated methods as they can be used in the presence of ambient light in the field and do not require a dark environment. More interestingly, chlorophyll fluorescence was found to be related to the amount of phenolic compound in the epidermis of leaves and fruit peels (Cerovic et al., 2002; Hagen et al., 2006; Agati et al., 2007). Anthocyanin, as an important part of phenolic compound, attracts much attention due to its antioxidant effects and health benefits (Khoo et al., 2017). Besides, anthocyanin content can help to assess quality, maturity stage, and also in determining the harvesting time of a plant (Christian and Jackson, 2009). The conventional destructive biochemical analysis of anthocyanin is discontinuous, time-consuming, and costly since it involves harvesting, multiple extraction steps before reaching the quantification stage (Nakata and Ohme-Takagi, 2014). Thus, there is a great demand for an advanced device that can be used for both PSA monitoring and fast non-destructive anthocyanin quantification to improve agricultural efficiency and productivity.

Nowadays, several fluorescence imagers and sensors are commercially available for non-invasive PSA monitoring, such as Walz Imaging-PAM, Photon Systems Instrument FluorCams, and LI-COR (Cen et al., 2017; van Tol et al., 2017; Wang et al., 2017; Backes et al., 2021; Linn et al., 2021). However, none of the above-mentioned imaging devices can be used to quantify anthocyanin content. Moreover, they are relatively bulky and expensive. Apart from the above mentioned devices, Force A founded in 2004 developed Dualex and Multiplex which have been used to quantify anthocyanin content through fluorescence emission (Hagen et al., 2006; Agati et al., 2007; Steele et al., 2009), but they are point sensors without imaging capability. Besides, they cannot quantitate plants’ PSA parameters that requires pulse-amplitude modulated (PAM) excitation. Hence, there is an unmet need for a compact and high-throughput fluorescence imager for multi-purpose applications. Technical challenges in developing such a system include combining multiple functions in single device, maintaining system performance, size, and cost reduction. Herein, we overcame these challenges and developed a compact handheld multifunctional and multi-spectral fluorescence imager with the following advantages: (1) it combines non-invasive and continuous PSA monitoring and anthocyanin distribution quantification in a single device; (2) it is a high-resolution imaging device, which inherently has high throughput measurement capability and can provide morphological information; (3) it is compact and low-cost by incorporating dedicated optoelectronics design. The study was divided into two parts to verify the functions of the self-developed device: PSA monitoring and non-invasive anthocyanin quantification. In the first part, the PSA of drought and control plants were measured by the imager to quantify the stress condition based on obtained fluorescence images. In the second part, non-invasive anthocyanin measurement was carried out and results were correlated with biochemical analysis to assess the quantification accuracy of our device.

## Materials and Methods

### Instrument Design

**FIGURE 1:**
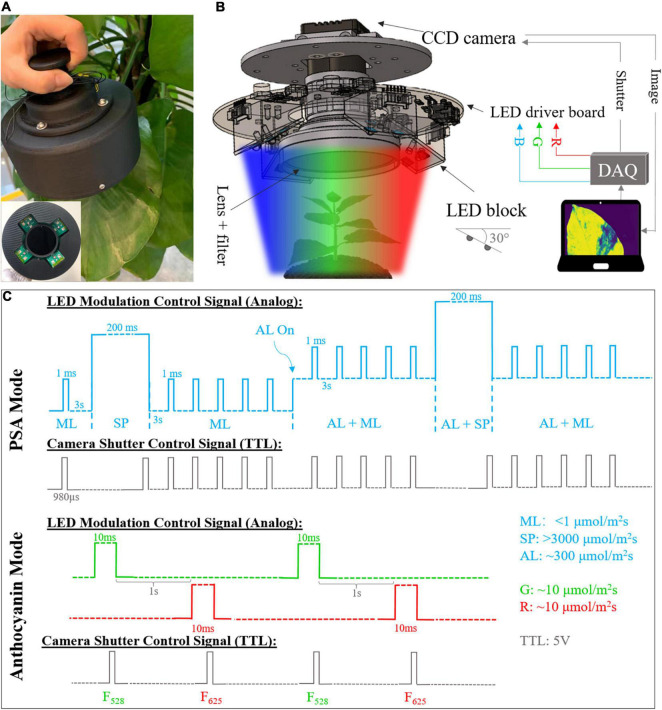
**(A)** Photograph of the developed compact fluorescence imager. Side view and front view (inset). **(B)** System diagram and inner structure of fluorescence imager. DAQ, data acquisition. **(C)** Timing diagram of light emitting diode (LED) modulation and camera shutter control signals for photosynthetic activity monitoring with blue LEDs. ML, measuring light; SP, saturation pulse; AL, actinic light; TTL, transistor–transistor logic. Note that the timing diagram is for illustration purpose only. The pulse widths and amplitudes are not presented in right scale due to limited space.

The developed compact fluorescence imager mainly consists of multi-spectral light emitting diode (LED panel, LED driver board, CCD camera with IMX273 sensor, imaging lenses, and filters. Its photo and overall schematic are shown in Figures 1A,B. The size reduction was achieved by using a customized board-level driver circuit and all-in-one multi-spectral LED (Cree XLamp XM-L) that provide high-intensity RGB output in a small package (5 mm × 5 mm). Each package contains 625 (± 5), 528 (± 8), and 458 (± 8) nm LED diodes and each of them can be controlled independently. A total of 12 RGB LEDs are connected in series and arranged in the groups of 3 on 4 LED blocks. Individual LED block was tilted by 30 degrees for better illumination. RGB color channels are controlled by three independent LED drivers (AP8802H, Diodes Incorporated). In this study, the LED was operated in PAM mode with different widths and amplitudes of pulse light to achieve dynamic illumination (Schreiber et al., 1986). The dimming of LEDs was adjusted by the 0–5 V analog output signal from the data acquisition (DAQ) card (USB-6003, NI). A board level mono color charge-coupled device (CCD) camera (A15S, Alkeria) was fixed in the center and employed as an image detector, together with an imaging lens and 695 nm long-pass filter (LPF). The working distance is adjustable from 25 mm to infinity and the resolution is 1,088 × 1,456.

### Fluorescence Data Acquisition and Processing

The device provides two operating modes, which can be used to monitor the PSA and measure anthocyanin content, non-invasively and continuously. Under PSA monitoring mode, the measuring light (ML), saturation pulse (SP), and actinic light (AL) in blue (465 nm) with different intensity and period are shined at a specific sequence. The modulation of various LED beams was controlled by analog output from DAQ card with different pulse widths and voltages. ML is a weak and short pulse used to measure the minimal fluorescence without causing any photosynthesis. It was set to be 1 ms with 3 s pulse interval and < 1 μmol photons m^–2^s^–1^. SP is a strong pulse that makes all photosystem II reaction centers closed, which is used to determine the maximal fluorescence yield. It was set at 200 ms with intensity of > 3,000 μmol photons m^–2^s^–1^. AL refers to the continuous light to drive the photosynthetic activity with intensity of ∼300 μmol photons m^–2^s^–1^. The illumination sequence of different pulses and the corresponding camera shutter signal are shown in Figure 1C. After 3-h dark adaption, the ML was applied to measure minimal fluorescence F_0_ pixel-wisely, followed by a SP to get maximal fluorescence F_*m*_. Then, 5 MLs were shined to measure the fluorescence response after SP. Note that the number of MLs is adjustable. The AL was turned on after that and together with sequential MLs and SPs, steady state fluorescence F (after photosynthesis is stable) and maximal fluorescence after light adaption F_*m*_’ were obtained. The camera shutter was fixed at 980 μs to acquire the images at the end of ML and SP. Fluorescence images were saved real-time for post-processing. The maximum efficiency and operating efficiency of photosystem II (PSII) for each pixel can be calculated as (Kitajima and Butler, 1975; Genty et al., 1989):


(1) $$F_{v}/F_{m}=\frac{F_{m}-F_{0}}{F_{m}}$$

(2) $$\phi_{\mathrm{PSII}}=\frac{F_{m}^{\prime }-F}{F_{m}^{\prime }}$$

For anthocyanin measurement, the green (528 nm) and red (625 nm) LEDs were shined sequentially with 10 ms pulse width and 1 s interval. The illumination intensity for both colors were adjusted and measured by a commercial photoactive radiation (PAR) meter (Apogee SQ-500) to ensure the same PAR output according to photosynthetic photon flux (Barnes et al., 1993). Anthocyanins are usually located in the epidermis of plant leaves to absorb light and protect plants from harmful radiation (Hughes et al., 2007; Zhu et al., 2018). The green and red LED induced images were acquired at the steady state. Thus, based on chlorophyll screening effect and Beer-Lambert Law, the pixel-wise anthocyanin index was estimated by the following formula (Agati et al., 2007):


(3) $$\mathrm{Anth}\,\_\,\mathrm{Index}=l\,o\,g\,\left(\frac{F_{625}}{F_{528}}\right)$$

where F_625_ and F_528_ are steady state fluorescence images excited by red and green LEDs.

### Plant Preparation for Photosynthetic Activity Measurement

**FIGURE 2:**
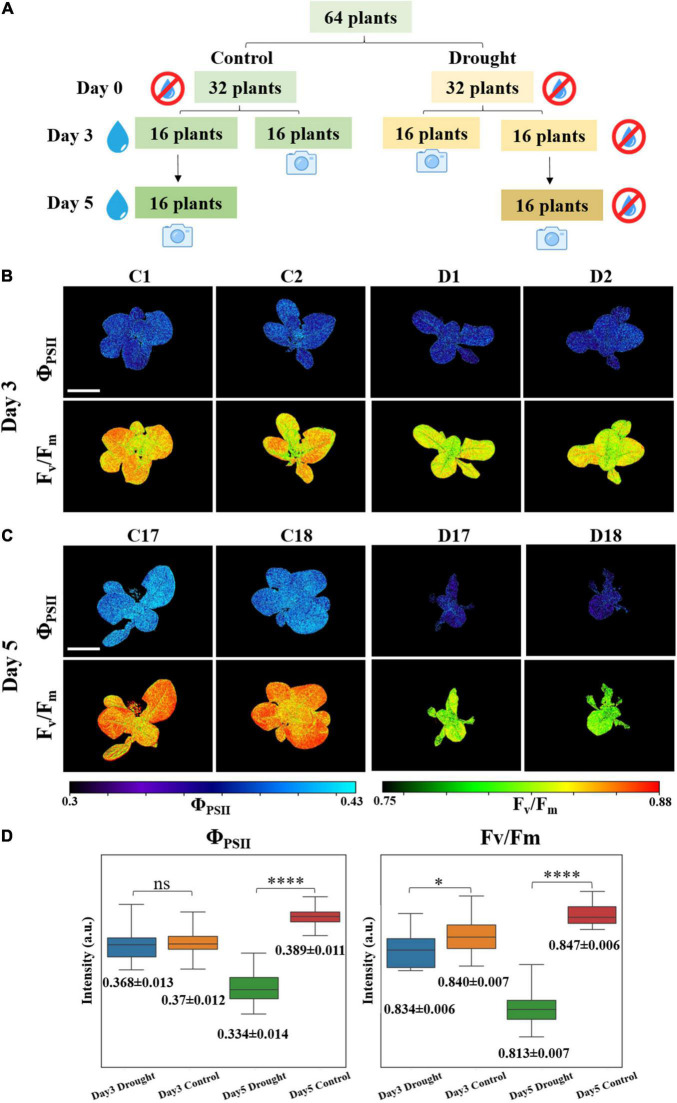
**(A)** Plants drought treatment for photosynthetic activity measurement. Examples of **(B)** day 3 and **(C)** day 5 fluorescence images of Φ_*PSII*_ and F_*v*_/F_*m*_. C represents for control group and D represents for drought group. The scale bar represents 5 cm. **(D)** Boxplots: mean values of 5,000 effective pixels randomly selected from Φ_*PSII*_ and F_*v*_/F_*m*_ images of the 16 plants in drought and control groups at days 3 and 5. Box is defined by 25th, 50th, and 75th percentiles. Top and bottom bars are maximum and minimum pixel intensities. Numbers below are mean values ± standard deviations (SDs). Student’s *t*-test were conducted to compare the difference of drought and control groups based on the values of *p* (*n* = 16, ns: *p* > 0.05, *p < 0.05, ^****^p < 0.0001).

Drought treatment was planned for PSA monitoring. Lettuce seeds (*Lactuca sativa* var. Little Gem) were surface sterilized using a 10% bleach solution containing Triton X-100, and then plated on Murashige and Skoog (1962) basal media with 1% agar. Plants were germinated and grown in a growth chamber at 22°C under 24 h continuous light and ∼90 μmol m^–2^ s^–1^ light intensity. After 4 days, lettuce seedlings were transferred to soil containing a 10:1 ratio of BVB peatmoss (BVB substrates, Netherlands) and sand. Plants were grown at 22 °C under a 16 h light/8 h dark cycle, ∼150 μmol m^–2^s^–1^ light intensity, and 70% relative humidity. Before drought treatment, all plants were watered daily to keep the soil wet. When the plants were 23 days old, watering was withheld. Then, 64 equally sized lettuce plants were randomly divided into control and drought groups. At days 3 and 5 after the start of drought treatment, water was added to control plants (till soil was saturated with water), and relevant non-invasive PSA measurements were taken in the afternoon about 4–6 h after watering. On day 3, 16 plants of each group were measured. The same procedure was repeated on day 5 and the remaining 32 plants of the two groups were measured. The whole process is illustrated in Figure 2A.

### Biochemical Analysis for Anthocyanin Content

For anthocyanin measurement, mixed salad leaves (Farmers’ Pick) were purchased from a supermarket. Then, 22 leaves including 5 green, 12 red, and 5 intermediate Batavia leaves (Supplementary Figure 1) were measured non-invasively by the developed fluorescence imager. Following non-invasive measurements, the biochemical quantification of total anthocyanins was carried out. The anthocyanin extraction was based on the procedure described by Neff and Chory (1998). The fresh weights of each sample were measured. Samples were placed in microfuge tubes and snap frozen in liquid nitrogen, then ground using a mortar and pestle. The extraction protocol is designed as follows where the volumes described in the following steps (except microplate reader measurements) were used for every 100 mg of fresh weight measured (total volumes were scaled according to the fresh weight of each sample). After grinding plant samples, 300 μl of methanol (containing 1% HCl) was added. Samples were incubated overnight in a dark refrigerator. On the next day, 200 μl water was added to each tube, followed by 500 μl chloroform. Then, samples were spun down in a centrifuge at 21,000 × *g* for 5 min. Furthermore, 400 μl of the supernatant (top methanol and water) phase was transferred into a new microfuge tube. Another 400 μl of 60% methanol (containing 1% HCl):40% water solution was added into the new microfuge tube. Two replicates of 200 μl per sample were pipetted in a 96-well microplate. Total anthocyanins were determined by measuring the A_530_ and A_657_ using a Spark multimode microplate reader (Tecan, Switzerland). Total anthocyanin content of the entire leaf sample in relative units was calculated based on the formula in Laby et al. (2000), except that the fresh weight was not divided:


$$\mathrm{Total}\,\mathrm{anthocyanins}=\left[O\,D_{530}-\left(0.25\times O\,D_{657}\right)\right]\times t\,o\,t\,a\,l\,e\,x\,t\,r\,a\,c\,t\,i\,o\,n\,v\,o\,l\,u\,m\,e\,\left(m\,L\right)$$

### Statistical Analysis

For the obtained Φ_*PSII*_ and F_*v*_/F_*m*_ fluorescence images, 5,000 pixels out of the non-zero pixels (effective pixels) for each plant were randomly selected and averaged. Background pixels with zero values were excluded. Then, the mean fluorescence intensities of the 16 plants in each group at days 3 and 5 were plotted in boxplots. The mean values and standard deviations (SDs) are calculated over the 16 plants for each group at each time point and summarized in Supplementary Table 1. The box was defined by the minimum, 25th percentile, median, 75th percentile, and the maximum of pixel intensities. Difference between the means of drought and control plants was compared by two-tail Student’s *t*-test using Python 3.7, SciPy toolbox.

Total anthocyanin index was calculated as the sum of every pixel’s value of anthocyanin image. Then, Random sample consensus (RANSAC), a robust regression algorithm was executed in Python to determine the correlation between non-invasively measured total anthocyanin index and destructive biochemical analysis result, as described in Equation 4. RANSAC can automatically exclude outliers during regression to enhance the overall predication accuracy. Data points whose residuals are out of the threshold (median absolute deviation (MAD) by default) are classified as outliers.

## Results

### High Throughput Plant Photosynthetic Activity Monitoring Under Drought Treatment

During PSA measurement, blue output from RGB LEDs was triggered and the fluorescence imager was fixed on the top of plants with fully opened aperture. The working distance was adjusted to ∼20 cm with the field of view (FOV) of ∼ 23 cm × 17 cm throughout the experiment to cover most part of the plants. The acquired series of fluorescence images were calculated pixel-wise based on Equations 1, 2 to obtain Φ_*PSII*_ and F_*v/*_F_*m*_ images on days 3 and 5. Representative images are shown in Figures 2B,C and the remaining images are provided in the Supplementary Figures 2, 3. From the images, the difference between control and drought groups can be clearly seen. At day 3, control group fluorescence images showed slightly higher values than drought group, especially for F_*v*_/F_*m*_. The difference between treatments became more pronounced at day 5. To better quantify these differences, statistical analysis mentioned in the section “Statistical Analysis” was performed and result is shown in Figure 2D and Supplementary Table 1. It can be calculated that at day 3, the Φ_*PSII*_ and F_*v*_/F_*m*_ mean values of control group are 0.46 and 0.61% higher than drought group, respectively. For Φ_*PSII*_, the value of *p* is 0.7 indicating that there is no statistically significant difference between the two groups. Nevertheless, F_*v*_/F_*m*_ appears to be a better indicator to detect plant stress at early stage, since *p* < 0.05 showing the difference between two groups is statistically significant. At day 5, mean values of Φ_*PSII*_ and F_*v*_/F_*m*_ increased by 5.3 and 0.96% for control group but decreased by 9.1 and 2.6% for drought group, with respect to day 3. Φ_*PSII*_ and F_*v*_/F_*m*_ of control group are 16.4 and 4.3% higher than drought group. Moreover, the values of *p* are almost 0 indicating that the stressed plants can be well distinguished at day 5 either by Φ_*PSII*_ or F_*v*_/F_*m*_. It is also worth noting that the spread, interquartile range (IQR), and SD of drought group at day 5 are larger than those in the control group, indicating that higher photosynthetic heterogeneity is present in the stressed plants. Successful identification of such heterogeneity by fluorescence imaging can assist in predicting plant response to environment (Bresson et al., 2015).

**FIGURE 3:**
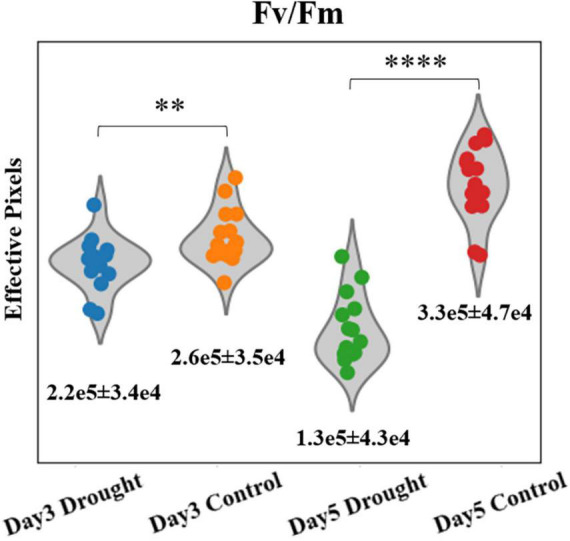
Violin plots of effective pixel numbers of F_*v*_/F_*m*_ images for drought and control plants at days 3 and 5 (^**^p < 0.01, ^****^p < 0.0001). Mean ± SDs are shown.

Unlike point sensors, which can only provide conventional photosynthetic parameters, such as Φ_*PSII*_ and F_*v*_/F_*m*_ etc., the homemade fluorescence imager can simultaneously provide morphological information to monitor plant canopy cover size using the single device, which is useful in studying the effect of stress on crop yields (Osborne, 2016; Anda et al., 2021). Since the whole plants’ canopies were imaged in the PSA experiment, the size can be easily quantified from any of the corresponding fluorescence images as an additional parameter. To estimate the plant size, we counted the number of effective pixels (non-zero) of F_*v*_/F_*m*_ images for individual plant, and are presented in Figure 3. The mean and SDs are summarized in Supplementary Table 2. At day 3 when water was added to the control group, the effective numbers of pixels of F_*v*_/F_*m*_ images is 18.1% higher for the control group, based on their mean values. The value of *p* of these two groups are 0.009. At day 5, the significant difference between drought and control groups can be seen. By counting the leaf area in F_*v*_/F_*m*_ images, the differences in mean effective pixels is ∼154%. The Student’s *t*-test of drought and control groups gave *p* < 0.0001 for F_*v*_/F_*m*_ images. Moreover, stressed plants decreased 41% in canopy size while control plants grew > 27% from day 3–5, according to F_*v*_/F_*m*_ effective pixels. The preliminary result proved that the fluorescence imager can provide both photosynthetic and morphological parameters simultaneously to monitor the plant condition, which is an advantage over normal cameras or point sensors.

### Non-invasive Anthocyanin Measurement vs. Biochemical Analysis

**FIGURE 4:**
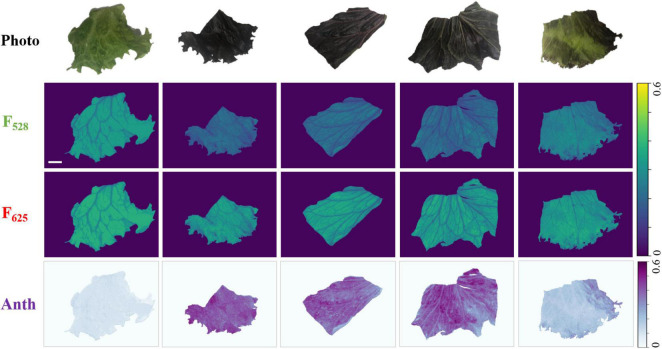
Representative images of the non-invasive anthocyanin measurement of green and red Batavia leaves. First row: the photos of sample leaves as reference. Second row and third rows: fluorescence images excited by 528 and 625 nm. Last row: the images of anthocyanin index (ANTH) which were calculated pixel-wisely from F_528_ and F_625_ based on Equation 3. The scale bar represents 1 cm.

A non-invasive anthocyanin measurement was conducted using red and green output from RGB LEDs as excitations. Working distance was adjusted to be ∼9 cm in this experiment and the corresponding FOV (8.5 cm × 6 cm) was enough to cover the entire leaf sample. Figure 4 shows the examples of non-invasive anthocyanin measurement done by the developed fluorescence imager. The first row shows photos taken by mobile phone camera as a reference. The second and third rows are the fluorescence images generated by 528 and 625 nm illumination, respectively. It should be noted that F_528_ and F_625_ are shown using the same colormap (viridis) and dynamic range for direct visualization and comparison. The anthocyanin distribution images calculated from F_528_ and F_625_ based on Equation 3 are drawn in Python BuPu colormap and shown in the last row. It can be clearly seen that for red Batavia, F_528_ is much lower than F_625_ resulting in the higher anthocyanin index. This is because anthocyanins absorb green light while transmits red light. More anthocyanins distributed in epidermis leads to the stronger attenuation of green light (Agati et al., 2007). Therefore, the amount of 528 nm photons that reached the mesophyll to excite chlorophyll fluorescence decreases. On the contrary, green Batavia leaf with extremely low or no anthocyanin content shows smaller difference between F_528_ and F_625_, since less green light is absorbed by the epidermis. The amount of red and green photons passing through epidermis to excite chlorophyll are similar, resulting in lower anthocyanin index, as expected. It can be considered that the anthocyanin index of green Batavia leaf is much lower than red one for every pixel, which is closer to the background signal. Hence, it looks light-colored and even “disappeared” in the anthocyanin distribution plot. Results for other samples can be found in Supplementary Figures 1, 4–6.

**FIGURE 5:**
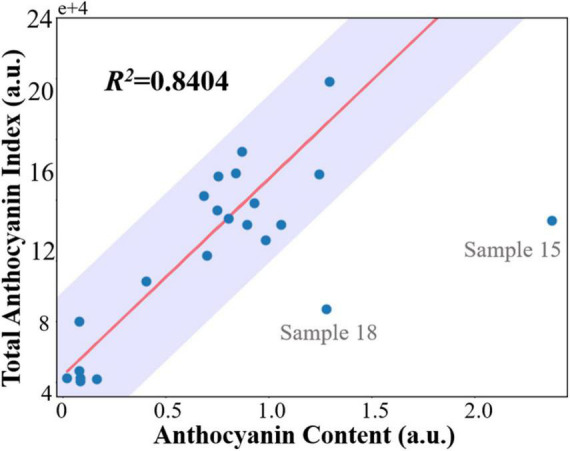
Correlation between the sum of pixel-wise anthocyanin index measured non-invasively by developed fluorescence imager (total anthocyanin index) and total anthocyanin content extracted from whole leaf. The biochemically measured anthocyanin content is expressed in arbitrary units. Data falling in the shaded area [residuals within median absolute deviations (MAD)] are classified as inliers.

To validate the non-invasive anthocyanin index measurement using a fluorescence imager, anthocyanin content from the leaf extracts was quantitated and correlated using a biochemical method. Results from biochemical measurements are presented in Supplementary Table 3. The regression and fitting were performed as described in section “Statistical Analysis.” The correlation results of destructive biochemical analysis and anthocyanin index are shown in Figure 5. Despite two outliers (sample 15 and 18) were spotted, the final coefficients of determination *R*^2^ are 0.8404 and 0.8420, respectively, indicating an excellent linear correlation and validating the effectiveness and accuracy of our compact fluorescence imager in the non-invasive anthocyanin measurement.

## Discussion

Our compact handheld fluorescence imager realized the non-invasive monitoring and quantification of PSA and anthocyanin content in plants and leaves, emphasizing its multifunctionality. First, with a series of blue pulse illuminations under PSA mode, the home-built fluorescence imager was able to continuously acquire fluorescence images and form fluorescence induction kinetic curves (Supplementary Figure 7), which reflect the plant transient response to the ambient light and energy transfer efficiencies. Φ_*PSII*_ and F_*v*_/F_*m*_ values were used to identify plant stress in drought experiment that showed similar values and trend as reported before (Wang et al., 2018; Shin et al., 2020). Apart from Φ_*PSII*_ and F_*v*_/F_*m*_ demonstrated in this work, more photochemical and non-photochemical quenching parameters can be also calculated from the curve (Maxwell and Johnson, 2000) that will be carried out in the planned future work. In addition, we demonstrated that plant canopy cover can be calculated from fluorescence images, so that both photosynthetic and morphological parameters can be obtained simultaneously, which makes it superior to normal cameras and fluorescence point sensors.

In addition, conventional destructive anthocyanin quantification by the biochemical analysis requires tedious procedures, which is time consuming and manpower intense. Our fluorescence imager with alternate red and green illuminations, can display anthocyanin content distribution fast and non-invasively in the FOV, for plant maturity stage determination (Ahmadiani et al., 2014) or quality evaluation. Zivcak et al. (2017) named fluorescence signal log(F_635_/F_516_) as ANTH index and they conducted a study to non-invasively quantify the total flavonoid content in lettuce using ANTH index measured by a single-point detector. They correlated it with total flavonoids measured by an aluminum chloride colorimetric method with quercetin calibration and obtained a coefficient of determination of *R*^2^ = 0.5356. In our study, log(F_625_/F_528_) was correlated with total anthocyanin content, the correlation was significantly improved to *R*^2^ = 0.84. Better correlation may owe to anthocyanin’s dominant absorption of red light among flavonoids. Additionally, thanks to its imaging capability, the entire leaf could be covered. Every pixel of leaf was counted, instead of a rough estimation based on a small area average. Supplementary Figure 8 shows correlation between average anthocyanin index and anthocyanin content per gram fresh weight (total anthocyanins/FW), where average anthocyanin index was calculated as the mean value of randomly selected 1,000 non-zero pixels for each sample. In this case, a power function was used for fitting, according to Agati et al. (2007) and achieved *R*^2^ = 0.8420. However, average anthocyanin index obtained by optical method is area based and expressed per pixel. On the other hand, biochemically-measured anthocyanin content is normalized to leaf weight and expressed per gram fresh weight. Hence, this correlation is the lack of validity without considering leaf fresh weight per area, which is not recommended. Overall, the imaging capability of our device will contribute to high throughput screening, detailed spatial heterogeneity detection and high spatial resolution etc., bringing in more convenience and possibilities for plant phenotyping and quality checks.

However, there are a few aspects that need to be considered in the operation of the system. First, the PAR illumination is only uniform at a certain distance, meaning that the leaves at different heights may undergo different illumination intensity. However, it will not affect the final result, since both PSA and anthocyanin measurement are based on the relative ratio or dynamic change of fluorescence signals during different illuminations. Second, for anthocyanin measurement, Equation 3 is only valid when anthocyanins are distributed in the epidermis of plants. Moreover, calibration curves may be different for different species. This could be addressed by calibrating the system with large datasets, which will be validated in our ongoing study in an indoor vertical farm. The purpose of this study was to introduce the tremendous capability of the system. In the future applications, the calibration and limit of detection need to be carried out and determined accordingly. Last, in the current study, a PC was used for real-time data transfer and processing. In future, powerful microcontroller and LCD screen will be incorporated for real-time on-board image processing and display, to make the fluorescence imager a stand-alone device toward commercialization.

## Conclusion

In conclusion, we have developed a multifunctional compact handheld fluorescence imager for non-invasive and high throughput plant phenotyping. Preliminary PSA measurement was conducted and plants under drought stress were successfully differentiated by photosynthetic and morphological indicators. Anthocyanin content in Batavia lettuce leaves was quantified non-invasively and showed good correlation with biochemical analysis. Being different from commercially available fluorescence imaging and sensing devices, our fluorescence imager utilizes all-in-one RGB LEDs, a dedicated driver circuit, and a method to achieve multifunction capability and compact size. To the best of our knowledge, it is the first-of-its-kind compact fluorescence imaging device for both the non-invasive PSA monitoring and anthocyanin distribution measurement. These features will help to significantly reduce manpower and cost in various applications to improve agricultural efficiency. We envision that this system has great potential in modern agriculture for both in-field and indoor farm settings.

## Data Availability Statement

The original contributions presented in the study are included in the article/Supplementary Material, further inquiries can be directed to the corresponding author/s.

## Author Contributions

RZ, UD, and DU conceived and designed the study. SK prepared the plant for PSA study and also carried out biochemical analysis. RZ, MT, and RB developed the device. RZ and MT conducted the fluorescence experiment. RZ and SK analyzed the data and wrote the manuscript. SZ and KD provided input and also revised the manuscript. DU, UD, and MO supervised the project. All authors contributed to the article and approved the submitted version.

## Conflict of Interest

The authors declare that the research was conducted in the absence of any commercial or financial relationships that could be construed as a potential conflict of interest.

## Publisher’s Note

All claims expressed in this article are solely those of the authors and do not necessarily represent those of their affiliated organizations, or those of the publisher, the editors and the reviewers. Any product that may be evaluated in this article, or claim that may be made by its manufacturer, is not guaranteed or endorsed by the publisher.
