# Supplementary material for: Handheld Multifunctional Fluorescence Imager for Non-invasive Plant Phenotyping
Source: Front Plant Sci. 2022 Apr 8;13:822634. doi: 10.3389/fpls.2022.822634 (PMC9024405; doi:10.3389/fpls.2022.822634)
Supplement: Supplementary file 1 [file Data_Sheet_1.PDF]

Supplementary Material

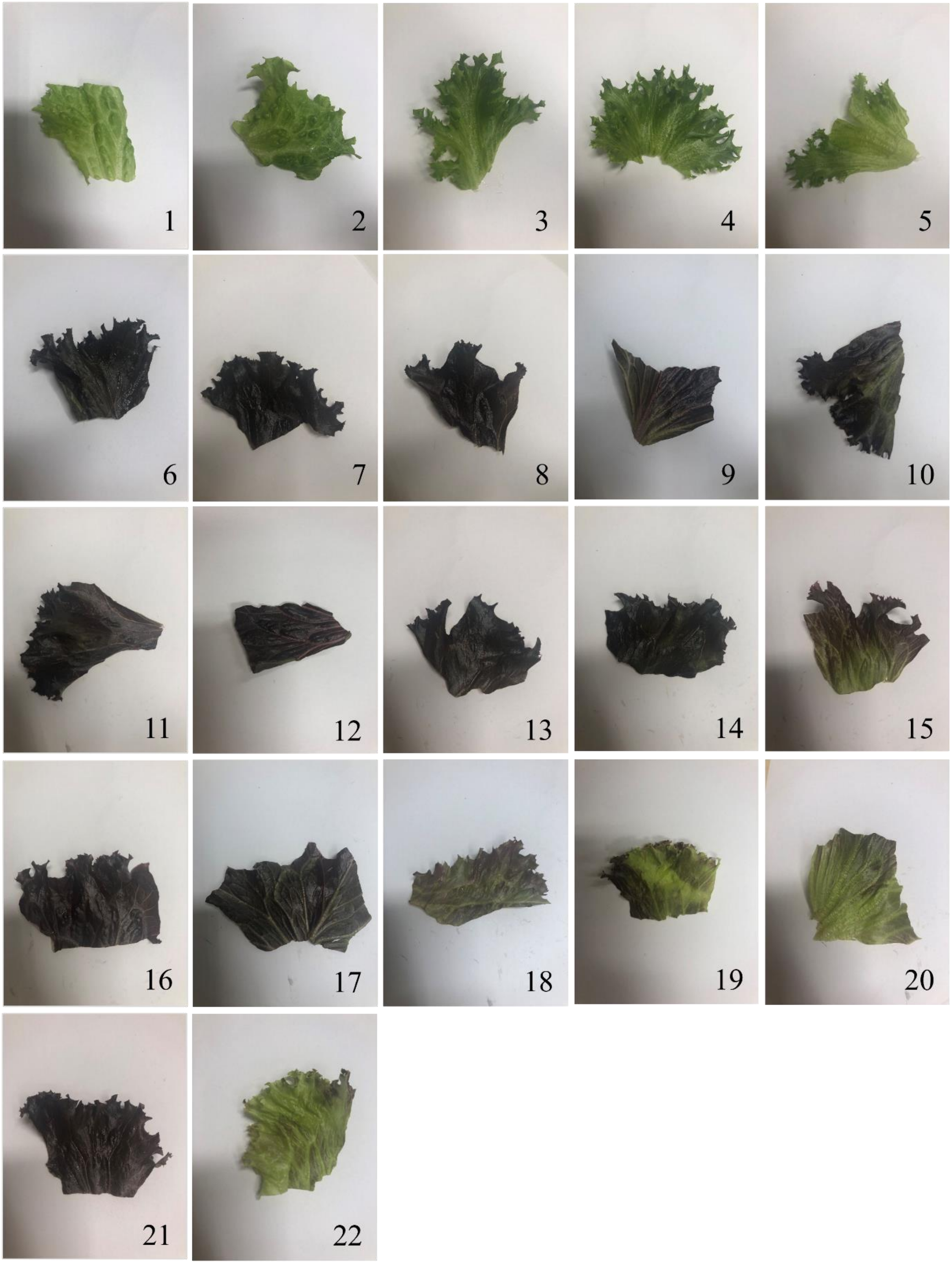

Supplementary Figure S1. Photographs of sample leaves used for noninvasive anthocyanin quantification.

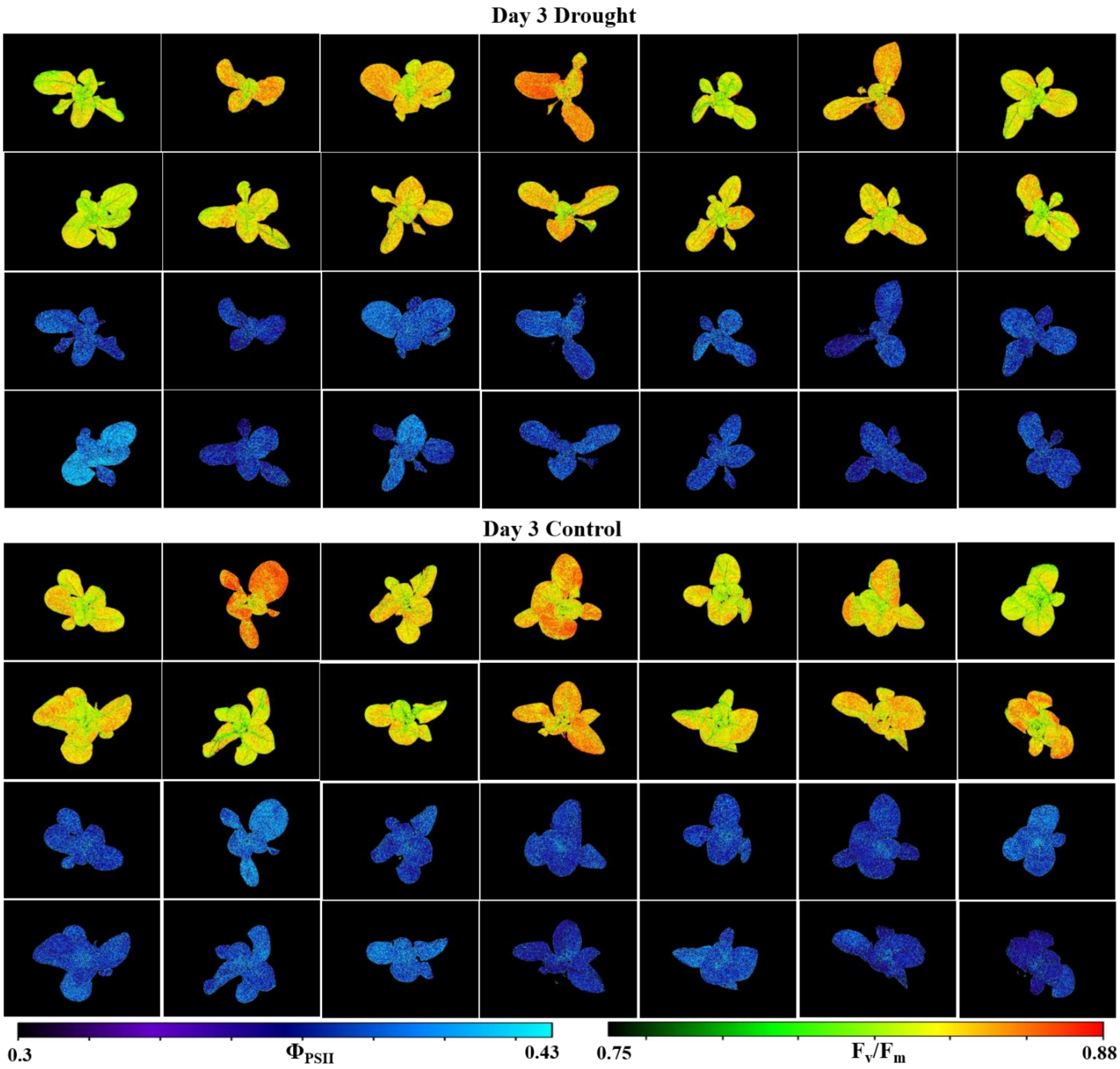

Supplementary Figure S2. Day 3 fluorescence images of Φ_{PSII} and Fv/Fm.

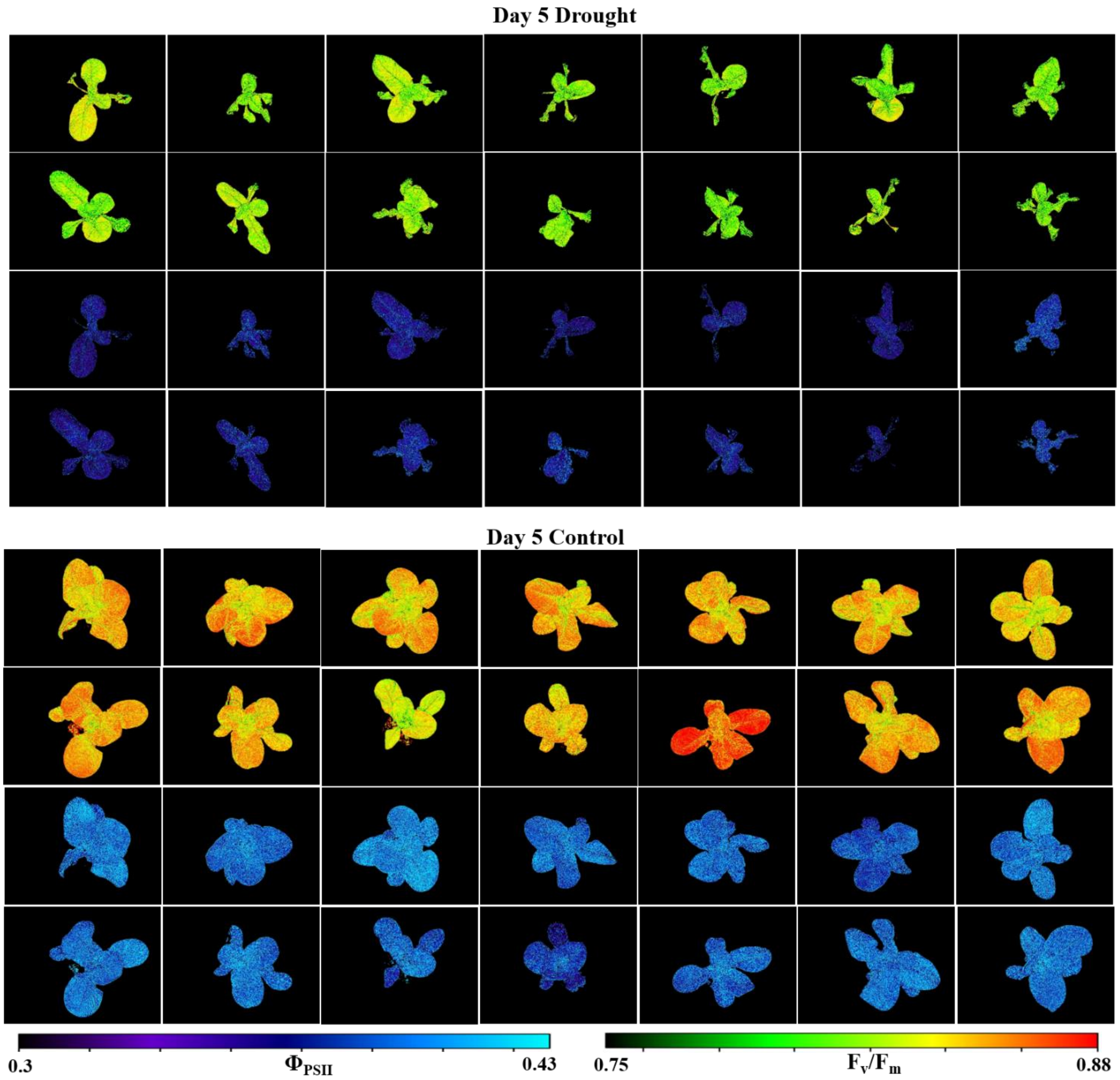

Supplementary Figure S3. Day 5 fluorescence images of Φ_{PSII} and Fv/Fm.

Supplementary Table S1. Summary of calculated mean and standard deviation of intensities from the obtained Φ_{PSII} and F_v/F_m images.

Intensity(a.u.)	Φ_{PSII}		F_v/F_m	
	Mean	Std	Mean	Std
**Day 3 Drought**	0.3678	0.0126	0.8344	0.0064
**Day 3 Control**	0.3695	0.0121	0.8395	0.0069
**Day 5 Drought**	0.3344	0.0142	0.8126	0.0070
**Day 5 Control**	0.3891	0.0114	0.8476	0.0059

Supplementary Table S2. Summary of calculated mean and standard deviation of effective pixels from the obtained F_v/F_m images.

Effective pixels	F_v/F_m	
	Mean	Std
**Day 3 Drought**	222982	33903
**Day 3 Control**	258201	35482
**Day 5 Drought**	130753	43253
**Day 5 Control**	330439	46810

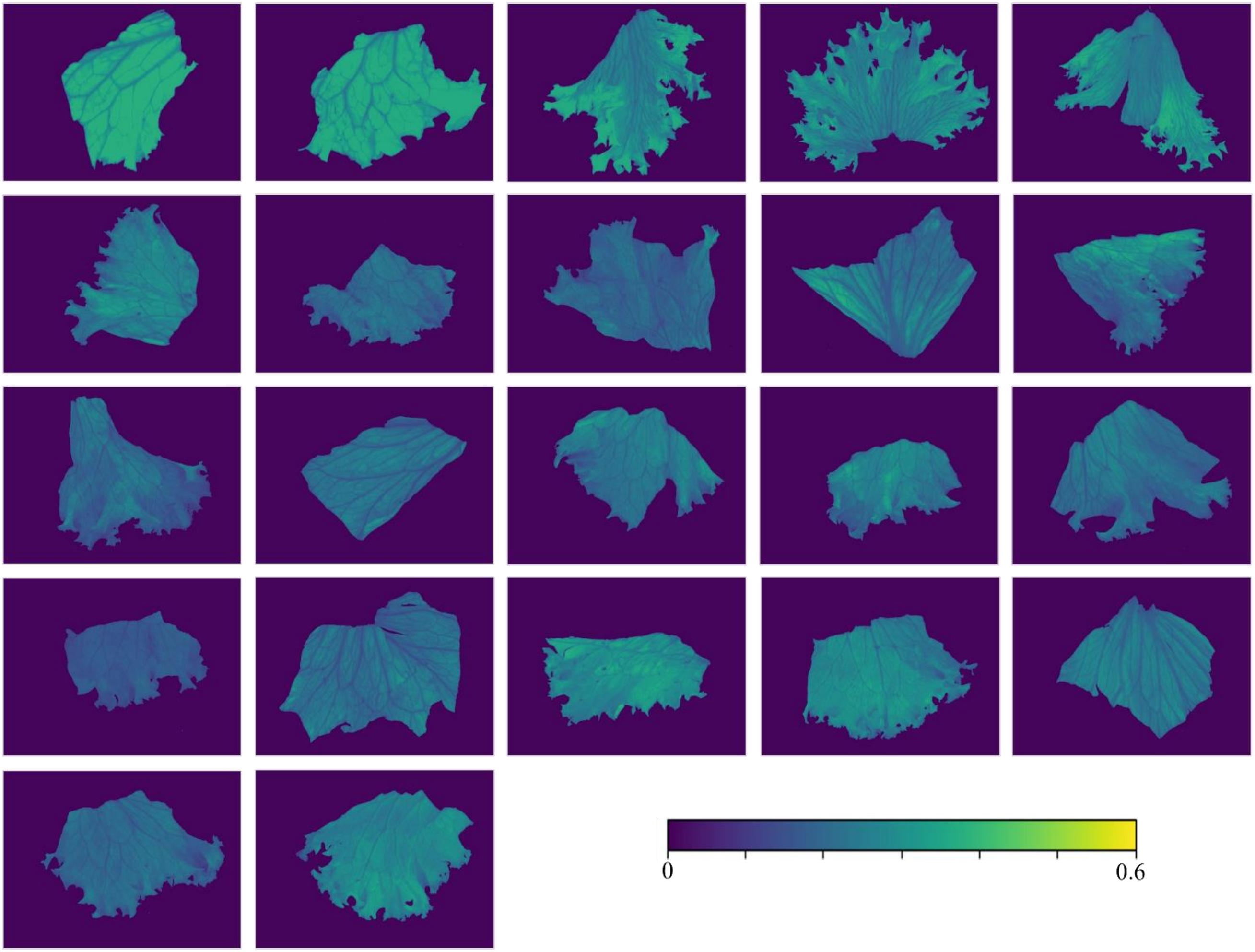

Supplementary Figure S4. Fluorescence images obtained at **528nm** excitation: **F_{528}**. Representative images are shown in Figure 4.

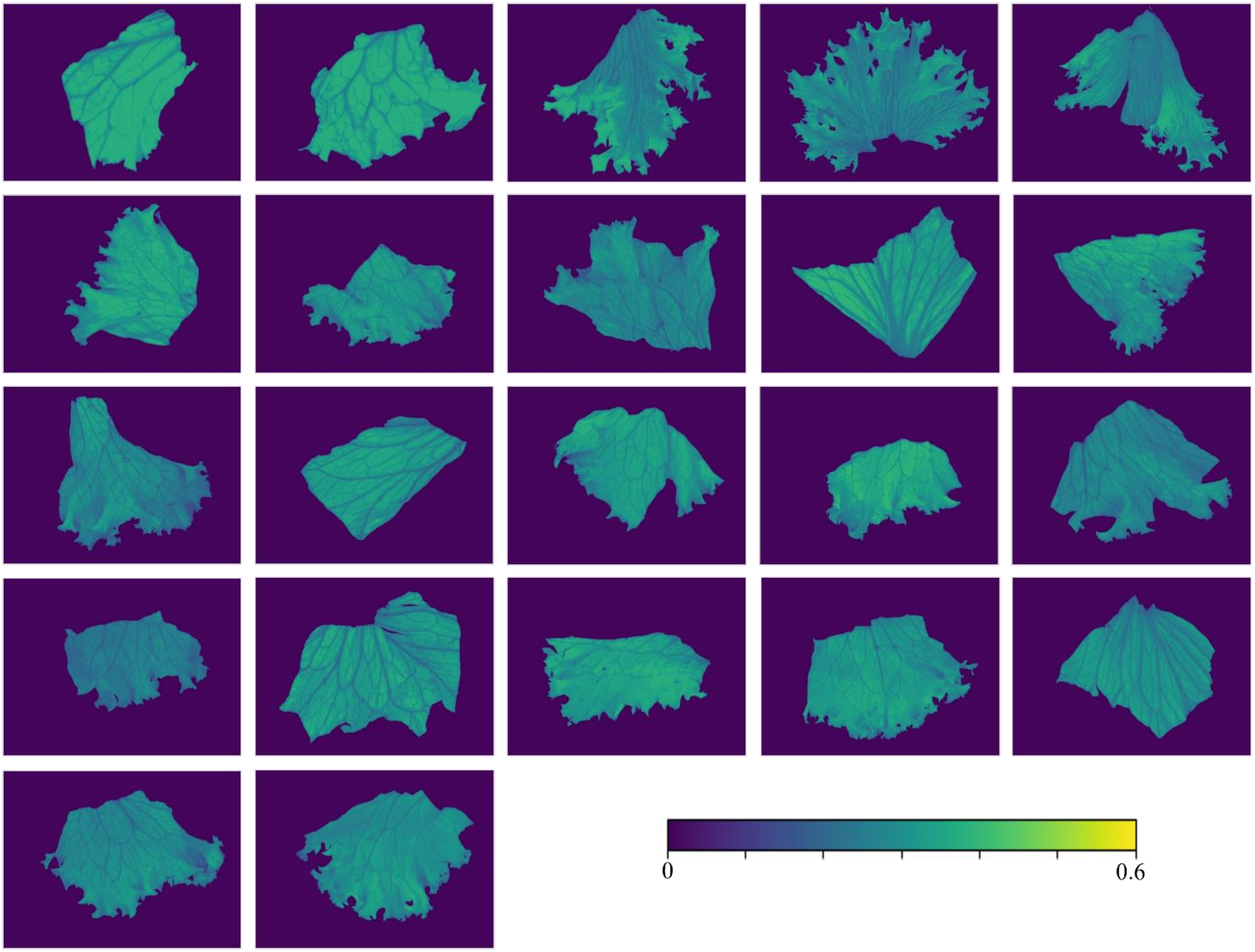

Supplementary Figure S5. Fluorescence images obtained at **625nm** excitation: $\mathbf{F_{625}}$. The colormap is the same with F_{528} in the same range for direct visualization and comparison. Representative images are presented in Figure 4.

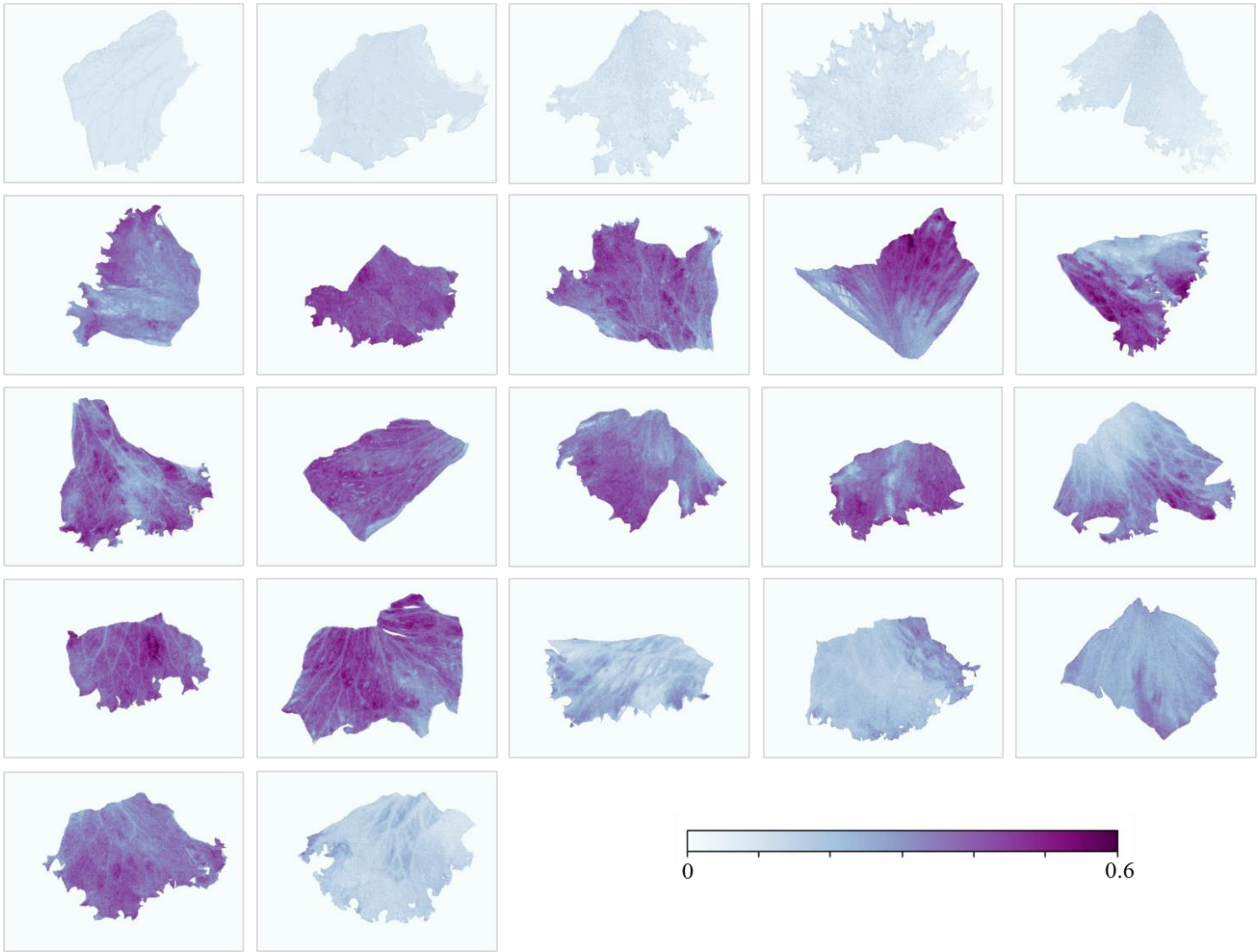

Supplementary Figure S6. Anthocyanin distribution calculated from $\mathbf{F_{528}}$ and $\mathbf{F_{625}}$ based on equation (3). Representative images are presented in Figure 4.

Supplementary Table S3. Biochemical analysis result of anthocyanin content in sample leaves.

Sample	Red/Green	FW (g)	Buffer (mL)	A530	A657	Anet	Anthocyanins_total
1	Green	0.7889	3	0.0166	0.0104	0.01400	0.084
2	Green	0.8613	3	0.01635	0.01085	0.01364	0.081825
3	Green	1.15	3	0.0172	0.01195	0.01421	0.085275
4	Green	1.4877	4.5	0.0225	0.0165	0.01838	0.165375
5	Green	1.7186	4.5	0.0032	0.0032	0.00240	0.0216
6	Red	0.4829	1.5	0.30075	0.01185	0.29779	0.8933625
7	Red	0.3271	1.5	0.2521	0.00615	0.25056	0.7516875
8	Red	0.6078	1.5	0.29515	0.0178	0.29070	0.8721
9	Red	1.1266	3	0.2113	0.0137	0.20788	1.24725
10	Red	0.4123	1.5	0.35555	0.01185	0.35259	1.0577625
11	Red	0.6081	1.5	0.28485	0.01785	0.28039	0.8411625
12	Red	0.6903	1.5	0.23175	0.01135	0.22891	0.6867375
13	Red	0.4419	1.5	0.31255	0.0069	0.31083	0.932475
14	Red	0.2995	1.5	0.32825	0.00135	0.32791	0.9837375
15	Intermediate	0.7488	3	0.39635	0.0037	0.39543	2.37255
16	Red	0.3189	1.5	0.26965	0.00495	0.26841	0.8052375
17	Red	0.9584	3	0.2151	-0.0027	0.21578	1.29465
18	Intermediate	0.3845	1.5	0.42435	-0.00725	0.42616	1.2784875
19	Intermediate	0.5567	1.5	0.1335	-0.0077	0.13543	0.406275
20	Intermediate	0.9702	3	0.11475	-0.00845	0.11686	0.701175
21	Red	0.4813	1.5	0.25285	0.0057	0.25143	0.754275
22	Intermediate	0.6755	1.5	0.0274	0.0032	0.02660	0.0798

A530 and A657 are measured values from Spark multimode microplate reader.

Anet=A530-0.25×A657

Anthocyanins_total = Anet×Buffer×2(two replicates)

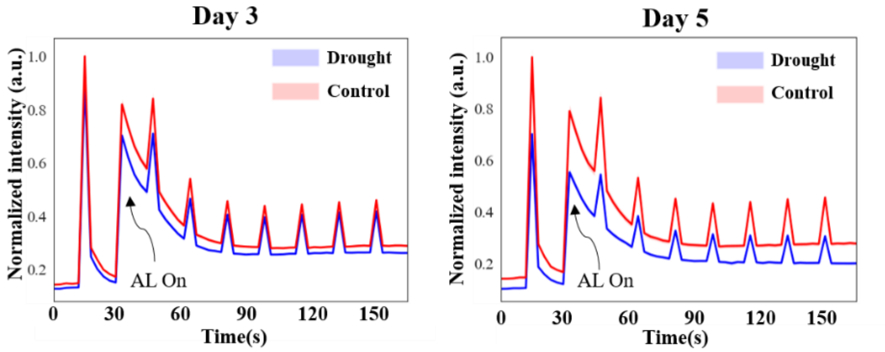

Supplementary Figure S7. Example of normalized fluorescence induction kinetics curves in drought and control conditions on Day 3 and Day 5. The curves are the average of the 16 plants in each group.

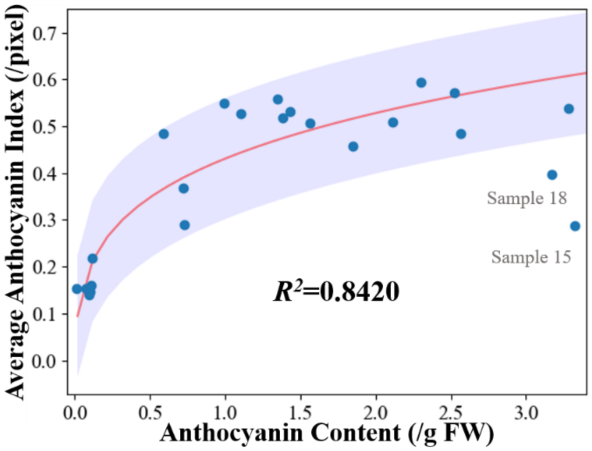

Supplementary Figure S8. Correlation between average anthocyanin index (mean value of 1000 effective pixels) and anthocyanin content normalized to fresh weight. Data falling in the shaded area are classified as inliers.
